# Intraoperative Nerve Monitoring Can Reduce Prevalence of Recurrent Laryngeal Nerve Injury in Thyroid Reoperations: Results of a Retrospective Cohort Study

**DOI:** 10.1007/s00268-013-2260-x

**Published:** 2013-10-01

**Authors:** Marcin Barczyński, Aleksander Konturek, Krzysztof Pragacz, Aleksandra Papier, Małgorzata Stopa, Wojciech Nowak

**Affiliations:** 1 Department of Endocrine Surgery, Third Chair of General Surgery, Jagiellonian University Medical College, 37 Prądnicka Street, 31-202 Kraków, Poland; 2 Department of General Surgery, Public Health Care Medical Center, 78 11 Listopada Street, 28-200 Staszów, Poland

## Abstract

**Background:**

The prevalence of recurrent laryngeal nerve (RLN) injury is higher in repeat than in primary thyroid operations. The use of intraoperative nerve monitoring (IONM) as an aid in dissection of the scar tissue is believed to minimize the risk of nerve injury. The aim of this study was to examine whether the use of IONM in thyroid reoperations can reduce the prevalence of RLN injury.

**Methods:**

This was a retrospective cohort study of patients who underwent thyroid reoperations with IONM versus with RLN visualization, but without IONM. The database of thyroid surgery was searched for eligible patients (treated in the years 1993–2012). The primary outcomes were transient and permanent RLN injury. Laryngoscopy was used to evaluate and follow RLN injury.

**Results:**

The study group comprised 854 patients (139 men, 715 women) operated for recurrent goiter (*n* = 576), recurrent hyperthyroidism (*n* = 36), completion thyroidectomy for cancer (*n* = 194) or recurrent thyroid cancer (*n* = 48), including 472 bilateral and 382 unilateral reoperations; 1,326 nerves at risk (NAR). A group of 306 patients (500 NAR) underwent reoperations with IONM and 548 patients (826 NAR) had reoperations with RLN visualization, but without IONM. Transient and permanent RLN injuries were found respectively in 13 (2.6 %) and seven (1.4 %) nerves with IONM versus 52 (6.3 %) and 20 (2.4 %) nerves without IONM (*p* = 0.003 and *p* = 0.202, respectively).

**Conclusions:**

IONM decreased the incidence of transient RLN paresis in repeat thyroid operations compared with nerve visualization alone. The prevalence of permanent RLN injury tended to be lower in thyroid reoperations with IONM, but statistical validation of the observed differences requires a sample size of 920 NAR per arm.

## Introduction

Thyroid reoperations can be challenging, even for a highly experienced thyroid surgeon, as visual identification of the recurrent laryngeal nerve (RLN) is more difficult during dissection of the scar tissues than in the virgin neck [1, 2]. The prevalence of RLN injury is higher in repeat than in primary thyroid operations and it has been reported to approach 12.5 % for transient events and 3.8 % for permanent events [1–11]. Unilateral RLN injury can diminish quality of life due to a variety of symptoms related to voice changes and subsequent limitations in physical, emotional and social functioning, while bilateral RLN injury can be a life-threatening complication leading to airway obstruction [12]. Thus, it is not surprising that RLN injury is still a major cause of litigation in endocrine surgery [13]. In recent years, intraoperative nerve monitoring (IONM) has been standardized and has gained more acceptance as an addition to the gold standard of visual nerve identification in thyroid and parathyroid surgery [14–18]. IONM is believed by many surgeons to aid in RLN dissection in the scar tissues of the neck, minimizing the risk of inadvertent nerve injury [14]. However, so far, this belief is not evidence based [11]. A prospective randomised controlled trial of RLN visualisation versus RLN visualisation plus IONM in redo thyroid surgery is not readily feasible, justifying the use of evidence from cohort studies. The aim of the present retrospective cohort study was to test the hypothesis that the use of IONM in thyroid reoperations can reduce the prevalence of RLN injury in comparison with thyroid reoperations with RLN visual identification alone.

## Materials and methods

This was a retrospective cohort study of patients who underwent thyroid reoperations at the Third Department of General Surgery, Jagiellonian University Medical College, Kraków, Poland. The prospectively collected database of thyroid surgery was searched for eligible patients (treated in the years 1993–2012). The study group comprised patients who underwent reoperative thyroid surgery with IONM. They were compared with patients who had thyroid reoperations with RLN visual identification, but without IONM. Laryngoscopy was mandatory in all patients before reoperation and afterwards, and was used to evaluate and follow RLN injury. All patients provided written informed consent for the storage and use of their data.

The inclusion criteria were recurrent goitre, recurrent hyperthyroidism, completion thyroidectomy for incidental cancer in a patient after bilateral less than near-total thyroidectomy (or Dunhill operation), or recurrent thyroid cancer. The exclusion criteria were contralateral goitre recurrence in a patient after lobectomy, completion thyroidectomy for cancer in a patient after lobectomy, goitre recurrence in the pyramidal lobe in a patient after total thyroidectomy or incomplete clinical data or follow-up information.

The primary outcome measures were transient and permanent RLN injury. The secondary outcome measures included the utility of IONM in mapping RLN in the scar tissues of the neck and in predicting postoperative vocal cord dysfunction: positive predictive value (PPV) and negative predictive value (NPV).

The protocol of this study was approved by the Institutional Review Board.

### Surgical technique

Surgeon-performed neck ultrasound was mandatory in all patients before reoperation. It was used to identify the dominant side of goitre recurrence (which was operated on first), to identify if the pyramidal lobe remnant was present and to evaluate thyroid remnant volume and lymph node status.

All operations were performed on patients under general anaesthesia by five experienced endocrine surgeons (MB, AK, SC, MS, WN). The anaesthesia protocol included intravenous midazolam premedication; induction with fentanyl, thiopental and suxamethonium; endotracheal intubation and sevoflurane maintenance. No other muscle relaxants were used during the operations. A standard cervicotomy with excision of the existing scar was performed in all patients. A lateral approach was routinely used for all cases. In operations without IONM, the first step was visual identification of the RLN low in the neck (below the crossing with the inferior thyroid artery). Once the nerve was visually identified, it was carefully dissected along its course towards the larynx. In operations with IONM, the visual identification of the RLN was facilitated via the IONM system, with the nerve mapping technique. Once the nerve was visually identified, repeated stimulations with the monopolar probe of the IONM system served to trace the nerve path in the operative field and test its functional integrity during dissection. In each patient, the RLN was exposed and the branches of the superior and inferior thyroid arteries were divided close to the thyroid capsule (peripheral ligation). The characteristics of the patients are presented in Table 1, while the extent of surgery is presented in Table 2.

**Table 1:** Characteristics of patients

Characteristics	With IONM	Without IONM
	*n* = 306	*n* = 548
Ratio (female:male)	247:59	440:108
Mean age ± SD (years)	54.6 ± 13.2	54.0 ± 13.6
Preoperative diagnosis		
Non-toxic multinodular goitre	220 (71.9)^†^	356 (65.0)^†^
Toxic multinodular goitre	9 (2.9)	17 (3.1)
Graves’ disease	2 (0.7)	8 (1.5)
Differentiated thyroid cancer	70 (22.9)^‡^	161 (29.4)^‡^
Medullary thyroid cancer	5 (1.6)	6 (1.1)
Number of previous operations		
1	258	485
2	29	59
3	1	4
Preoperative RLN palsy λ	7 (2.3)	11 (2.0)

Data are presented as numbers (%) unless otherwise indicated. λ calculated for patients

*IONM* intraoperative nerve monitoring, *RLN* recurrent laryngeal nerve, *SD* standard deviation

^†^ *p* = 0.038; ^‡^ *p* = 0.040 all other differences were not significant

**Table 2:** Extent of surgery

	With IONM (*n* = 306)	Without IONM (*n* = 548)	*p* value
NAR	500	826	NA
Bilateral/unilateral dissections (ratio)^a^	194/112 (1.7)	278/270 (1.0)	<0.001
Surgical intervention			
Total thyroidectomy	178 (58.2)	154 (28.1)	<0.001
Near-total thyroidectomy	12 (3.9)	56 (10.2)	0.001
Bilateral subtotal thyroidectomy	4 (1.3)	68 (12.4)	<0.001
Lobectomy	110 (35.9)	201 (36.7)	0.831
Subcapsular lobectomy	2 (0.7)	69 (12.6)	<0.001
Central neck dissection	31 (10.1)	48 (8.8)	0.507
Lateral neck dissection	16 (5.2)	27 (4.9)	0.847
Staged thyroidectomy in case of LOS λ	23 (7.5)	Not used	NA
Completion of staged thyroidectomy in case of LOS during first reoperation λ	11 (3.6)	Not used	NA

Data are presented as numbers (%)

λ calculated for patients. χ^2^ test for all

*IONM* intraoperative nerve monitoring, *LOS* loss of signal on the dominant side (which was operated on first), *NA* not applicable, *RLN* recurrent laryngeal nerve

^a^Patients with preoperative RLN paresis were calculated as unilateral dissections if contralateral side of the neck was operated on

### IONM technique

During this study period, two differing neuromonitoring systems were used at our unit. The Neurosign^®^ 100 system (Inomed, Teningen, Germany) was in use between 2004 and 2007, while the NIM^®^ 2.0 followed by the NIM^®^ 3.0 (Medtronic, Jacksonville, USA) was in use between 2008 and 2012. The Neurosing^®^ 100 system operated with needle electrodes. After identification of the cricoid and thyroid cartilage, the ipsilateral vocal muscle was impaled with the bipolar recording electrode through the cricothyroid ligament. The neutral electrode was placed in the sternocleidomastoid muscle. The proper placement of the electrodes was confirmed by an impedance meter of the circuit in the patient in the final operating position. A hand-held bipolar, concentric stimulating probe was used with a current amplitude of 1 (range 0.5–1.0) mA (depending on the RLN threshold) and 3-Hz impulses of 200 ms each for 1–2 s. The electrical field response of the muscle was documented as an acoustic signal. The NIM^®^ system operated with surface electrodes integrated with an endotracheal tube 7.0–7.5 in diameter, which was inserted by an anaesthetist between the vocal folds under direct vision during intubation. The standardized technique of neuromonitoring of the RLNs was used, including indirect vagal response evaluation at the beginning and at the end of the operation (IONM = L1 + V1 + R1 + R2 + V2 + L2) according to the recommendations formulated recently by the International Intraoperative Neural Monitoring Study Group [14]. The nerves were stimulated using a monopolar electrode and the interrupted stimulation technique at 1 mA, 100 ms impulse duration and 4 Hz frequency. In case of bifurcated RLN nerves, the assessment included post-stimulation response of each nerve branch based on acoustic evaluation of the signal, EMG response evaluation (latency and amplitude) and the technique of posterior larynx palpation (‘laryngeal twitch’). After the removal of the thyroid lobe, the indirect stimulation (through the electrode placed on the ipsilateral vagus nerve) response was determined and used to predict the postoperative outcome.

The validity of nerve monitoring was defined and calculated according to Chan and Lo [19]. A missing signal was considered positive, predicting postoperative ipsilateral vocal cord injury. The test result was regarded as true positive when ipsilateral RLN paresis was confirmed on a postoperative laryngoscopic examination and false positive when normal ipsilateral vocal cord function was found. An intact signal after the thyroidectomy was considered negative, predicting normal postoperative vocal cord function. This was interpreted as true negative if there was normal ipsilateral vocal cord function and false negative if there was a postoperative laryngoscopic diagnosis of RLN paresis.

### Perioperative management and follow-up

Laryngoscopy (either indirect or direct videolaryngoscopy) by a throat specialist was mandatory before surgery and on day 1 after surgery. In patients with RLN paresis, an additional examination was scheduled at 1, 2, 4, 6 and 12 months after surgery, or until the vocal cord function was recovered. Vocal cord paresis for more than 12 months after the operation was regarded as permanent palsy.

### Statistical analysis

For a sample size calculation, an assumption was made that the use of IONM as an addition to the gold standard of visual identification of the RLN should reduce the prevalence of RLN injury by 50 % or more. To detect this, it was calculated that 492 nerves at risk (NAR) would be required in each treatment arm to give the study a power of 80 % with *p* = 0.05 to reliably evaluate RLN injury reduction from 6 to 3 %. The incidence of nerve events was calculated based on the number of NAR. Data are presented as mean (standard deviation) or mean (range), unless stated otherwise. The statistical significance of categorical variables was evaluated using the χ^2^ test, whereas the Students *t* test was used for the analysis of continuous variables. All data were collected prospectively, stored in a computer-based institutional register of thyroid surgery and analysed retrospectively by a statistician, assuming that *p* < 0.05 was indicative of significance. Statistical analyses were performed with Statistica^®^ 10 for Windows^®^ (StatSoft, Kraków, Poland).

## Results

**Fig. 1:**
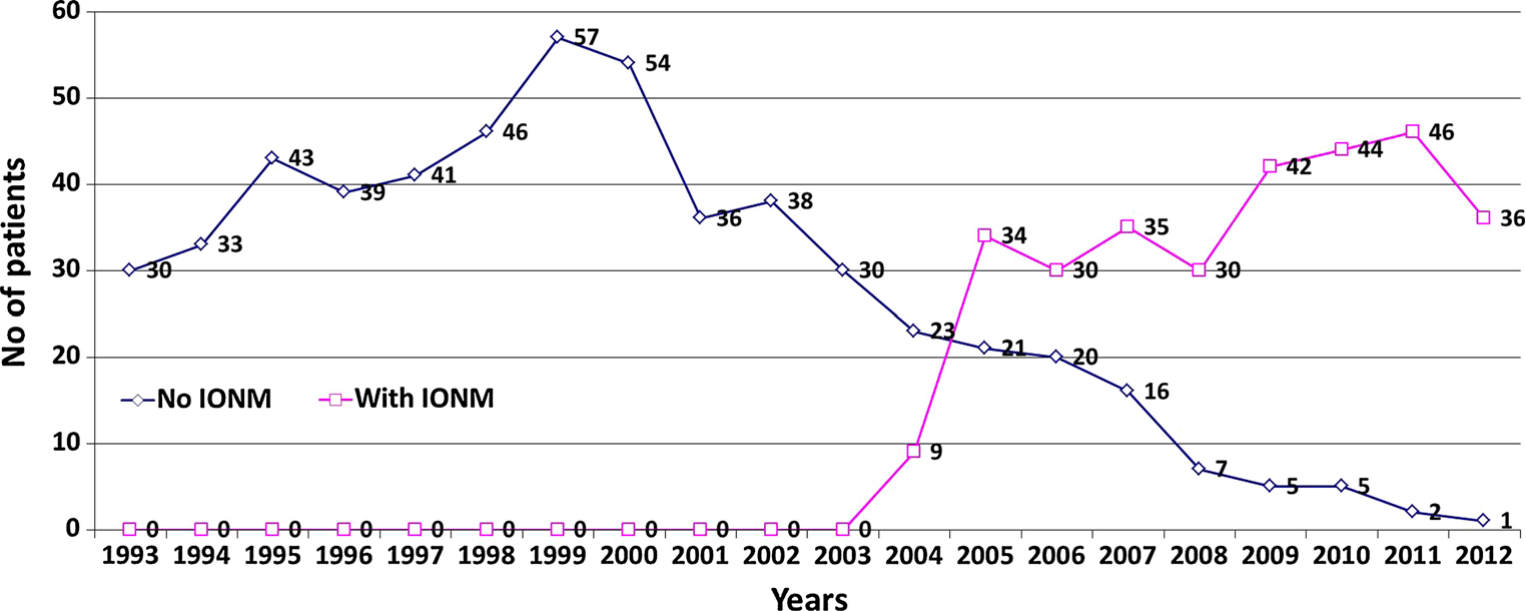
Year-to-year distribution of thyroid reoperations with respect to use of IONM among patients treated in 1993–2012. No IONM (*n* = 548), with IONM (*n* = 306)

Of 15,383 patients referred for thyroid surgery during the study interval, 1,151 underwent reoperative thyroid surgery and were potential candidates for the study. A total of 297 patients did not meet the inclusion criteria (completion thyroidectomy for cancer in a patient after lobectomy 161; contralateral goitre recurrence in a patient after lobectomy 87; incomplete clinical data 23 or follow-up information 19; goitre recurrence in the pyramidal lobe in a patient after total thyroidectomy 7), leaving 854 patients who were finally included in this study. There were 715 women and 139 men, with a mean age at diagnosis of 54.2 ± 13.4 years. The IONM system became available at our unit in 2004 and, since then, it has been utilized in both primary and reoperative thyroid surgery with increasing frequency (Fig. 1). The study group comprised 306 patients (194 bilateral and 112 unilateral reoperations; 500 NAR) who underwent reoperative thyroid surgery with RLN visualization and IONM. The Neurosign^®^ 100 system (Inomed, Teningen, Germany) was used in reoperations of 108 patients treated in 2004–2007, while the NIM^®^ 2.0 followed by the NIM^®^ 3.0 (Medtronic, Jacksonville, USA) was used in reoperations of 198 patients treated in 2008–2012. A group of 548 patients (278 bilateral and 270 unilateral reoperations; 826 NAR) who had thyroid reoperations with RLN visualization but without IONM served as the control group. A clinical characteristics of the patients analysed in this study is shown in Table 1; the extent of surgery is shown in Table 2. Both median and mean operative time was shorter for reoperations performed with versus without IONM (80 and 88.3 ± 23.3 min vs. 100 and 94.5 ± 23.0 min; *p* < 0.001).

### Primary outcome measures analysis

In operations with RLN visualization plus additional IONM, the prevalence of RLN injury, transient RLN paresis and permanent RLN palsy was, respectively, 4.7 % (*p* = 0.001), 3.7 % (*p* = 0.003) and 1.0 % (*p* = 0.202) lower than with visualization alone. At 12-month follow-up, 52 of 72 patients (72.2 %) having visualization alone versus 13 of 20 (65.0 %) individuals having additional IONM recovered their vocal cord function (median 4 months after surgery; range 2–12 months after surgery). Details are shown in Table 3. No bilateral RLN palsy occurred in patients reoperated with IONM, but it was a sequel necessitating tracheostomy in one patient with pre-existing contralateral RLN palsy who underwent reoperation without IONM (0 vs. 0.2 %, respectively; *p* = 0.455).

**Table 3:** Incidence of RLN injuries

Postoperative laryngoscopy	NAR		*p* value
	With IONM (*n* = 500)	Without IONM (*n* = 826)	
Paresis			
Transient	13 (2.6)	52 (6.3)	0.003
Permanent	7 (1.4)	20 (2.4)^a^	0.202
Total paresis	20 (4.0)	72 (8.7)^a^	0.001

Data are presented as numbers (%)

χ^2^ test for all

*IONM* intraoperative nerve monitoring, *RLN* recurrent laryngeal nerve

^a^Bilateral RLN palsy occurred in one patient with pre-existing contralateral RLN palsy who underwent reoperation without IONM

### Secondary outcome measures analysis

Utilization of the nerve mapping technique allowed for localization of 97 (19.4 %) of the RLN nerves before visual nerve exposition and almost twofold more ramified nerves than RLN visualization alone: 99/826 (12.0 %) versus 107/500 (21.4 %); *p* < 0.001. In addition, the non-RLN was identified in 9 of 500 NAR (1.8 %) operated with IONM versus 4 of 826 (0.5 %) NAR operated without IONM; *p* = 0.02.

The value of IONM in predicting postoperative vocal cord function was encouraging. The following predictive values of the vagus stimulation (V2) at the end of surgery were found for the entire cohort of 500 NAR operated with IONM: NPV 99.6 %; PPV 78.3 %; and overall accuracy 98.6 %. Detailed data related to loss of signal predictive values are presented in Table 4. In our experience, the NIM 2.0 or NIM 3.0 systems had similar NPV and overall accuracy as the Neurosign^®^ 100 system (99.7 vs. 99.4 % and 99.4 vs. 97.0 %, respectively), but better PPV (90.9 vs. 66.7 %, respectively).

**Table 4:** Correlation of IONM results with postoperative outcomes

IONM^a^	Postoperative outcome (n)		Total (*n*)	Predictive value	%
	RLN paresis	No RLN paresis			
LOS (positive)	18	5	23	PPV	78.3
Intact signal (negative)	2	475	477	NPV	99.6
Total	20	480	500	Accuracy	98.6

Operations were performed with Neurosign^®^ 100 (*n* = 108) and NIM 2.0 or NIM 3.0 systems (*n* = 198)

*Accuracy* TP + TN/TP + TN + FP + FN, *IONM* intraoperative nerve monitoring, *LOS* loss of signal, *RLN* recurrent laryngeal nerve, *PPV* positive predictive value (TP/TP + FP), *NPV* negative predictive value (TN/TN + FN), *TP* true positive, *TN* true negative, *FP* false positive, *FN* false negative

^a^Calculated for indirect stimulation through vagus nerve after thyroid resection (V2) and for NAR

## Discussion

IONM has gained widespread acceptance as an adjunct to the gold standard of visual nerve identification, and this technique can be used to identify the RLN and the external branch of the superior laryngeal nerve (EBSLN) in both primary thyroid surgery and thyroid reoperations [11, 14, 15, 18, 20–23]. The nerve may be mapped out in the operative field before visual identification, and additional intermittent stimulation of adjacent non-neural tissue versus nerve can help in tracing the nerve and all its branches through the dissected field. In addition, electric nerve testing at the end of the operation can serve for postoperative prognostication of nerve function [14]. In a prospective, randomized study of 1,000 patients (1,000 NAR per arm) published in 2009 by Barczyński et al. [17] the prevalence of transient RLN paresis was significantly lower in the group of patients who had primary thyroid surgery with IONM (by 2.9 % in high-risk patients and by 0.9 % in low-risk patients). The rate of permanent nerve palsy tended to be lower in operations performed with versus without IONM (by 30 %), but the power of this study was not sufficient to validate this difference. A more recent study by the same authors published in 2012 showed that the use of IONM significantly improved the identification rate of the EBSLN during thyroidectomy, as well as reduced the risk of early phonation changes after thyroidectomy [22, 23].

A reoperation in the scar tissue of the central neck is more dangerous for the RLN (and also for the parathyroid glands) than a primary operation, and the utilization of IONM is believed to aid in redo dissection, which should minimize the risk of nerve injury. It is well known that the prevalence of RLN injury after thyroid reoperations is variable (details are shown in Table 5) and dependent on surgeons’ experience, and hence it is lower in high-volume centres [8, 24, 25]. Despite the fact that thyroid reoperations have become less common due to a wide acceptance of total thyroidectomy as a standard treatment for benign thyroid disease in tertiary referral centres, one-half of thyroid surgery worldwide is performed in low-volume centres, in which subtotal thyroidectomy is still a common procedure [25]. In recent years, the majority of patients undergoing thyroid reoperations in our unit were referred from outside low-volume institutions; *n* = 215 of 306 patients (70.3 %) undergoing surgery with IONM in 2004–2012. Referring physicians recognized both the experience with and the availability of the IONM system at our institution as a guarantee of redo thyroid surgery being performed as safely as possible. However, so far, this belief is not entirely evidence based. It is well known that experience and routine nerve exposure are crucial to minimization of RLN complications [1, 2, 24, 25]. However, the IONM-added value in reducing RLN-related morbidity in the reoperative setting remains unclear. Yarbrough et al. [20] employed IONM in 52 cervical re-exploration procedures and found that it could be performed safely, but did not decrease RLN morbidity. Alesina et al. [11] recently reported a retrospective series of 250 thyroid reoperations, including 91 operations performed with IONM, and 159 surgeries with direct nerve visualization but without IONM, and no significant differences were observed in this study in the prevalence of RLN injury between both groups. Nevertheless, the outcomes of these studies could be influenced by selection bias, as decision to use IONM was dependent on the availability of the equipment and individual surgeon’s preferences according to the planned extent of the surgical procedure (a more liberal use of IONM in more challenging dissections). On the other hand, these studies were based on relatively small groups of patients, and statistical validation of small differences in the prevalence of RLN injury requires much bigger study arms.

**Table 5:** RLN injury after reoperative thyroid surgery in published English-language series larger than 100 patients

Author	Year	Patients (*n*)	NAR (*n*)	Temporary RLN injury (%)	Permanent RLN palsy (%)
Reeve et al. [1]	1988	408	nd	6 (1.5)^a^	nd
Levin et al. [2]	1992	114	nd	1 (0.9)^a^	1 (0.9)^a^
Seiler et al. [3]	1996	166	242	nd	10 (4.1)
Chao et al. [4]	1997	115	nd	3 (2.6)^a^	2 (1.7)^a^
Menegaux et al. [5]	1999	202	nd	5 (2.5)^a^	2 (1.0)^a^
Müller et al. [6]	2001	949	1,307	46 (3.5)	33 (2.5)
Gibelin et al. [7]	2004	122	nd	15 (12.3)^a^	1 (0.8)^a^
Lefevre et al. [8]	2007	685	nd	8 (1.2)^a^	10 (1.5)^a^
Calò et al. [9]	2012	106	174	5 (2.9)	1 (0.6)
Kurmann et al. [10]	2012	109	133 ipsilateral	nd	5 (3.8)
			33 contralateral	nd	0 (0)

RLN injury without vs. with IONM			Without vs. with IONM	Without vs. with IONM	Without vs. with IONM
Alesina et al. [11]	2012	246	161 vs. 128	4 (2.5) vs. 8 (6.2)	1 (0.6) vs. 0 (0)
Present study	2013	836	826 vs. 500	52 (6.3) vs. 13 (2.6)^‡^	20 (2.4) vs. 7 (1.4)

Data are presented as numbers (%) unless otherwise indicated

Calculation for NAR unless otherwise indicated

*IONM* intraoperative nerve monitoring, *nd* no data, *RLN* recurrent laryngeal nerve

^‡^ *p* = 0.003

^a^Calculation for patients, not for NAR

As indicated by the results of our power calculation, 492 NAR are required in each treatment arm to give the study a power of 80 % with *p* = 0.05 to reliably evaluate RLN injury reduction from 6 to 3 %. The present study is the largest comparative analysis of thyroid reoperations performed with versus without IONM. It is important to stress that the prevalence of transient RLN paresis was decreased by almost 60 % in operations with RLN visualization plus additional IONM than RLN visualization alone (2.5 vs. 6.3 %, respectively; *p* = 0.003). In addition, the prevalence of permanent RLN palsy was 40 % lower in reoperations with IONM than with RLN visualization alone, but this difference was statistically not significant (*p* = 0.202). Given the outcomes of this study, a sample size of 920 NAR per arm was calculated as required to reliably assess the difference in the prevalence of permanent RLN events.

The explanation of better outcomes achieved in thyroid reoperations with IONM versus RLN visual identification alone should include a few important issues. First, IONM provided improved recognition of the anatomical details in the scar tissues of the neck with the use of the nerve mapping technique (almost 20 % of the RLNs were identified with IONM before visual exposition of the nerve, and almost twofold more ramified nerves were identified in reoperations with IONM). In addition, the rare but particularly prone to injury non-RLN was almost fourfold more often identified in patients operated with versus without IONM (*p* = 0.02). A lack of the initial vagus response (V1) when stimulating below the inferior thyroid artery, but positivity when stimulating above the inferior thyroid artery in a patient with intact preoperative ipsilateral vocal cord mobility, and a latency of the response shorter than 3.5 ms may alert the surgeon to the occurrence of this rare variant of the nerve before any surgical dissection is undertaken. This technique is of paramount importance in avoiding inadvertent and in most cases unrecognized intraoperative injury to the non-RLN [26, 27]. Second, intermittent RLN stimulation during the operation allowed for functional testing of the nerve. In case of EMG amplitude decrease during the case amounting to a drop of more than 30–50 % from the baseline value, even if the RLN seemed to be anatomically intact, surgeons stopped the dissection for a while and continued it with reduced traction of the nerve, observing in most cases a spontaneous EMG signal recovery. Third, in case of a loss of signal on the dominant side of the neck, which was, as a rule, operated on first (defined as no EMG signal at all, or a weak EMG waveform amplitude of less than 100 μV after ipsilateral vagus stimulation), the contralateral thyroid reoperation was staged for both benign (*n* = 20) and malignant disease (*n* = 3). Contralateral thyroid surgery was undertaken afterwards when ipsilateral vocal fold mobility was restored (*n* = 11). Two patients refused to undergo completion thyroidectomy after staged procedure for loss of signal. Details are shown in Tables 2, 3 and 4. In general, contralateral thyroid surgery was avoided in patients with benign thyroid disease and the only functioning RLN (permanent palsy), but it was undertaken in selected patients with contralateral loco-regional recurrence of thyroid cancer. The concept of staged thyroidectomy was proven to be effective in preventing bilateral RLN injury [28, 29], which is particularly important in bilateral thyroid reoperations and has been accepted by many [18, 30], but not all, thyroid surgeons [31]. The opponents of staged thyroidectomy indicate that PPV of IONM is relatively low (varying from 50 to 75 % in the majority of reported series), which can result in unnecessary staging of operation in 25–50 % of patients who experience loss of signal on the first side, but have no ipsilateral RLN paresis [31]. In this study, PPV of IONM was within the upper limits of the reported values (see Table 4), and it was better for the systems based on EMG waveform analysis (90.9 %) than for simple nerve stimulators with acoustic signalling (66.7 %). Thus, further improvements in PPV can be expected with utilization of continuous IONM, based on automatic permanent stimulation of the vagus nerve and almost real-time monitoring of the EMG quantitative parameters during surgery [32]. It should be also recognized that a cost-effectiveness analysis of the routine utilization of IONM in thyroid reoperations has never been reported and was also out of scope for this study; this issue needs to be clarified in future.

In conclusion, in our hands, IONM decreased the incidence of transient RLN paresis in repeat thyroid operations as compared with nerve visualization alone. The prevalence of permanent RLN injury tended to be lower in reoperations with IONM. However, given the outcomes of this study, a sample size of 920 NAR per arm is required to reliably assess a difference in the prevalence of permanent RLN events. Experience and routine nerve exposure remain crucial to the minimization of RLN complications in thyroid reoperations, but the use of the lateral approach with mapping of the RLN low in the neck and tracing its path in the scar tissues towards the larynx may provide additional benefits: an improved recognition rate of the ramified or non-recurrent nerves, and functional RLN testing feedback alerting the surgeon to a possible stretching injury of the nerve, particularly in the region of the ligament of Berry [33].
